# Prediction of Optimal Conditions of Hydrogenation Reaction Using the Likelihood Ranking Approach

**DOI:** 10.3390/ijms23010248

**Published:** 2021-12-27

**Authors:** Valentina A. Afonina, Daniyar A. Mazitov, Albina Nurmukhametova, Maxim D. Shevelev, Dina A. Khasanova, Ramil I. Nugmanov, Vladimir A. Burilov, Timur I. Madzhidov, Alexandre Varnek

**Affiliations:** 1 Chemoinformatics and Molecular Modelling Lab, A.M. Butlerov Institute of Chemistry, Kazan Federal University, Kremlyovskaya Str. 18, 420008 Kazan, Russia; ValAAfonina@kpfu.ru (V.A.A.); DaniAMazitov@kpfu.ru (D.A.M.); albinka2491@mail.ru (A.N.); mdshev7@gmail.com (M.D.S.); DAHasanova@stud.kpfu.ru (D.A.K.); nougmanoff@hotmail.com (R.I.N.); Vladimir.Burilov@kpfu.ru (V.A.B.); 2 Laboratory of Chemoinformatics (UMR 7140 CNRS/UniStra), Université de Strasbourg, 4, Rue Blaise Pascal, 67000 Strasbourg, France; 3 Institute for Chemical Reaction Design and Discovery (WPI-ICReDD), Hokkaido University, Kita 21 Nishi 10, Kita-ku, Sapporo 001-0021, Japan

**Keywords:** chemoinformatics, reaction informatics, ranking, artificial neural networks, QSAR, condensed graph of reaction, reaction conditions

## Abstract

The selection of experimental conditions leading to a reasonable yield is an important and essential element for the automated development of a synthesis plan and the subsequent synthesis of the target compound. The classical QSPR approach, requiring one-to-one correspondence between chemical structure and a target property, can be used for optimal reaction conditions prediction only on a limited scale when only one condition component (e.g., catalyst or solvent) is considered. However, a particular reaction can proceed under several different conditions. In this paper, we describe the Likelihood Ranking Model representing an artificial neural network that outputs a list of different conditions ranked according to their suitability to a given chemical transformation. Benchmarking calculations demonstrated that our model outperformed some popular approaches to the theoretical assessment of reaction conditions, such as k Nearest Neighbors, and a recurrent artificial neural network performance prediction of condition components (reagents, solvents, catalysts, and temperature). The ability of the Likelihood Ranking model trained on a hydrogenation reactions dataset, (~42,000 reactions) from Reaxys^®^ database, to propose conditions that led to the desired product was validated experimentally on a set of three reactions with rich selectivity issues.

## 1. Introduction

Nowadays, an interest in automation is growing every year, particularly in the field of chemistry where the creation of a fully automatized robochemist has become realistic [1,2,3,4,5]. One of the problems of the automated development of a synthesis plan of the target compound is the selection of experimental conditions leading to a reasonable yield. Earlier, various theoretical approaches, ranging from quantum chemical methods [6] to an artificial neural network of complex architecture [7], were used for condition assessment. These studies were mostly focused on predictions of the role a compound as a condition component: solvent; catalyst; or reagent. Thus, Struebing et al. [6] predicted solvents that enhanced kinetics of the Menshutkin reactions based on quantum chemical rate-constant calculations coupled with linear free energy relationship methodology. Marcou et al. [8] reported machine-learning models that could predict solvent and catalyst classes built on a hand-crafted Michael additions dataset of 222 reactions. Lin A. et al. [9] used the similarity-based approach to predict a general catalyst class for deprotection under catalytic hydrogenation conditions. Segler and Waller [10] used a knowledge graph built on a big reaction database complemented with chemical reasoning in order to predict an optimal combination of reagents and catalysts. Walker et al. [11] reported k-Nearest Neighbors, support vector machines and artificial neural networks models that predicted solvent or catalyst for Diels-Alder, Friedel-Crafts, Wittig, Aldol addition, and Claisen condensation. Unlike the above mentioned models that predict only one selected component of condition components, Gao et al. [7] reported a deep neural network that recurrently predicted the catalysts, reagents, solvents, and temperature. Although this model was trained on a large dataset of ~10 million reactions, it did not consider pressure due to a lack of data. To sample a set of conditions for a given reaction, the top-three reagents and solvents were predicted along with the top-two catalysts, top-one co-solvent and co-reagent, thus giving rise to their 18 combinations. The roles of additives and their names were taken from Reaxys^®^ as is, without any specific cleaning. At the same time, both Lin et al. [9] and Walker et al. [11] pointed out that the names of the compounds and their role (reactant, reagent, catalyst) require additional curation due to a significant number of duplicates and errors in the data. Nonetheless, performance of the Gao et al. model [7] was quite reasonable: for the test set reactions, experimentally used catalysts, reagents, and solvents were found in 57% top-ten predictions.

The most challenging problems facing a reaction condition prediction are the sparsity of reaction-condition data, absence of negative results, and many-to-many correspondence between chemical transformations and related conditions. The problem of sparsity originates in the fact that the same reaction can potentially be carried out under different conditions and only a few of them were really tested. The fact that predicted conditions are not found in the reaction database for a particular transformation does not necessarily mean that this prediction is wrong: the reaction might proceed under these conditions, however the latter simply were never tested experimentally.

The problem of the absence of negative results is caused by the lack of information on conditions, under which a given transformation cannot proceed. Such experimental data are rarely published, thus they are not available for modelling. Therefore, there is no way to perform negative control based on the existing data. The same problem arises with information retrieval domains regarding the prediction of the relevance of a particular web-page to a user query. There is no way to find non-relevant pages, i.e., negative examples. For this reason, in this article (and similarly to information retrieval domain) we decided to use a ranking approach, which seems to be the most natural solution to this condition prediction complexity issue. Noticeably, the classical QSPR approach requiring a one-to-one correspondence between the chemical structure and a target property can hardly be applied to the condition prediction due to the many-to-many correspondences between conditions and transformations. This can only be used in a limited scale, when one condition component (e.g., catalyst or solvent) is predicted at once, as we have seen in the abovementioned studies.

In this paper we describe a new approach to predicting a list of possible conditions, ranked according to their suitability, to a given chemical transformation. The conditions, under which the transformation has more of a chance to proceed, are expected to be at the top of the list. Since exact correspondence between the predicted and the experimental condition is not required, such a task is not sensitive to the absence of both negative results and a full list of suited conditions. Below, we report the condition ranking model based on an artificial neural network capable of simultaneously predicting temperature, pressure, catalyst, and the type of additive. In order to assess the prediction performances of the model, both null and baseline (ranking k Nearest Neighbors approach) models as well as quality metrics were suggested. The proposed approach was tested on hydrogenation reactions widely used in chemical synthesis and particularly important for medicinal chemistry [12]. The products of these reactions are very sensitive with respect to the choice of temperature, pressure, catalyst and additive, and therefore the prediction of their conditions represents a real challenge for the modelers.

## 2. Results and Discussion

The approaches for ranking possible reaction conditions were proposed and implemented. Likelihood ranking model (LRM) is a feed-forward neural network that ranks conditions on the basis of their predicted likelihood. k Nearest Neighbour (kNN) model returns suitable conditions in the order of corresponding k nearest reactions from training set. In principle, kNN reflects the reasoning of human chemists upon the selection of optimal conditions on the basis of available experimental data. These approaches were compared with null model, ASKOS model [7] and the model inspired by the one used in Gao et al. [7] (hereafter RecNN). Details of the model implementations and benchmarking criteria are described in Section 3.

**Figure 1:**
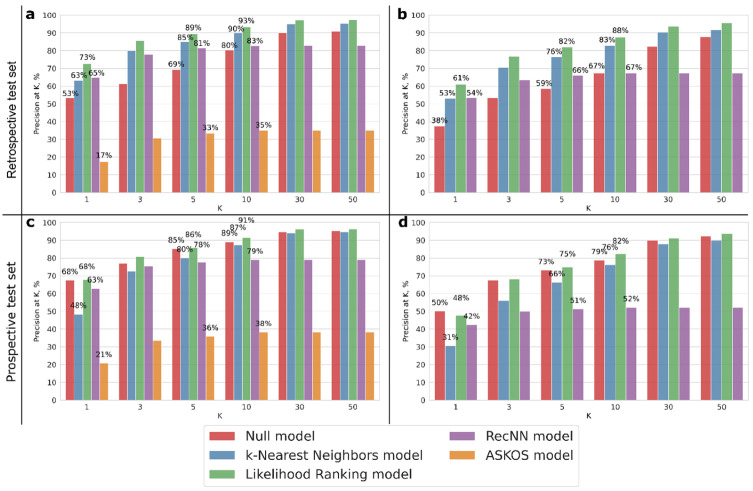
Cumulative histogram of the P@K on test sets vs. K; (**a**,**b**)—retrospective test set, (**c**,**d**)—prospective test set; (**a**,**c**)—pressure is ignored.

The best neural network architecture (found by hyperparameters optimization with MRR@5 metric) of LRM had a single hidden layer with 2000 neurons and ReLU activation function, trained in 200 epochs. At the first stage, LRM was benchmarked against existing models. Since the ASKOS model could not predict the pressure, this model was ignored at this step. The LRM, kNN, and RecNN models were trained on the training set containing by ca 35,000 reactions. The predictive performance of these approaches and the ASKOS model trained on the entire Reaxys^®^ dataset [7] was assessed on the retrospective test set using the P@K metric (Figure 1a).

As presented in Figure 1a, the null model is already good enough—the most common conditions were used in 53% of cases in the test set. The test set includes 37.5% reactions proceeding under palladium on carbon, medium temperature, and low pressure, and 13.65% reactions proceeding under palladium on carbon, medium temperature, and medium pressure. LRM demonstrated the best results: experimentally observed condition components combination turned out to be the first in the list of predictions (K = 1) in 73% of the cases. The kNN model also shows reasonable results: the difference between LRM and kNN model performances gradually decreases from 10% on the first rank to 4% with K = 5.

Surprisingly, the ASKOS model [7] performance is pretty bad: P@K varies from 17% (TOP-1) to 35% (TOP-10). This can be explained by the fact that the ASKOS model was trained on a large data set including various types of reactions, and not just catalytic hydrogenation reactions. Thus, the model was not biased to a particular reaction type. Also, as reported in the original publication [7], the training set of this model included 87.3% reactions without a catalyst. This explains why the ASKOS model tends to predict conditions without any catalyst: for 233 reactions from the retrospective test set and 226 reactions from the prospective test set, no catalyst was suggested. At the same time, only catalytic hydrogenation reactions are considered in our training and test sets and, therefore, other models always predict some catalyst. Moreover, ASKOS returns a maximum of 18 predictions, thus the percent of experimental conditions in the predictions of this model is stable after 20 TOP ranks.

For a fairer comparison with the Gao et al. approach [7], we implemented the RecNN architecture described by Gao et al., but adapted this to a given problem by matches architecture to the representation of the conditions in our dataset, and trained on our training set. Also, we used the same enumeration procedure of the condition components combination as in the original publication [7]. Enumeration of all allowed condition component combinations led to a drastic increase in the computational time due to the recurrent nature of the model. As shown in Figure 1b, compared to the ASKOS model, RecNN significantly improved the prediction accuracy from 17% to 54% in the TOP-1 predicted conditions and from 35% to 67% in the TOP-10 predicted conditions. On the other hand, RecNN performs slightly worse compared to LRM and the kNN, but better than the null model. To sum up, despite its relatively simple architecture, the LRM demonstrated a significantly better predictive performance compared to the benchmarked deep learning architectures.

Furthermore, the models were compared taking into account pressure predictions (Figure 1b). In this case, the trend described above was reproduced: LRM displayed the best results slightly outperforming the kNN model; the RecNN performed worse than kNN, yet still better than the null model. The difference between the LRM and kNN performances is about 5–8%. Performance metrics in 5-fold cross-validation carried out on the training set for LRM, kNN, and the null model are almost the same as in the retrospective validation (see Figure S7 in Supplementary Materials).

The trend observed for the prospective set is similar (Figure 1c,d), however the gap between the performances of RecNN and the null models significantly decreases. This could be explained by the high performance of the null model, which is slightly outperformed by LRM only. The former is related to the relatively small diversity of the prospective set: it contains only 144 unique combinations of condition components, which is almost twice as small as in the retrospective set (264 combinations). Also, more common catalysts were used more frequently in the prospective test set than in the retrospective and training set. For example, one can notice greater P@K for the null model in the prospective test set. Thus, poorer performance on the prospective test set does not necessarily mean that the model is getting bad. Simply, this may not fit a human preference considered as a “ground truth”, whereas other possible predictions are, in principle, feasible despite never being experimentally tested.

Table 1 shows how often particular conditions are reproduced in top-K proposed condition combinations. One can notice that the prediction of any particular condition components always outperforms the null model. This means that our approach extracts some hidden knowledge from the data. Catalyst predictions were quite accurate, achieving 98% for the top-10 considered combinations. Temperature and additives were predicted much more accurately than pressure. This could be explained by some skewing of the pressure annotation: most reactions (56.37% of the training set) proceed under atmospheric pressure.

Accounting for the model Applicability domain (AD) slightly improved the model performances, although this did not significantly change the trend reported in Figure 1 (see Figure S8 in Supplementary Materials).

Thus, the relatively simple Likelihood Ranking Model outperforms other tested approaches including deep neural networks. LRM slightly outperforms the kNN model, which represents quite a widespread heuristic that similar reactions proceed in the same or similar conditions [9].

**Figure 2:**
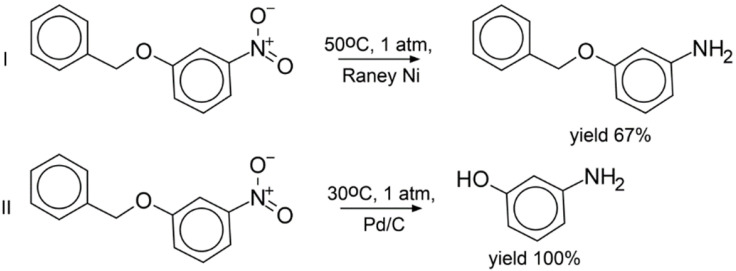
Regioselective reduction of 1-benzyloxy-3-nitrobenzene. Reaction I: predicted conditions were medium t, low p, unknown nickel catalyst; yield of target product was 67%, 33% was the initial compound. Reaction II: predicted conditions were medium t, low p, palladium on carbon; yield of target product was 100%.

**Figure 3:**
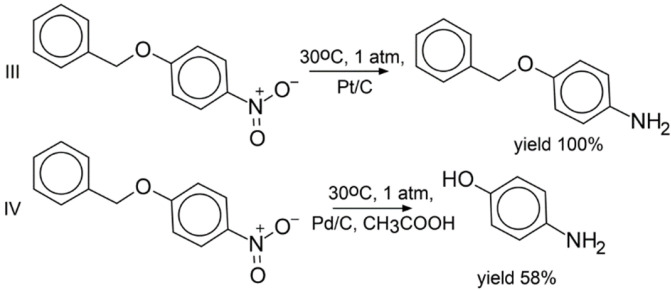
Regioselective reduction of 1-benzyloxy-4-nitrobenzene. Reaction III: predicted condition combination was medium t, low p, platinum on carbon; yield of target product was 100%. Reaction IV: predicted conditions were medium t, low p, palladium on carbon, acid; yield of target product was 58%, 42% was the incomplete reduction product—4-(benzyloxy)aniline.

**Figure 4:**
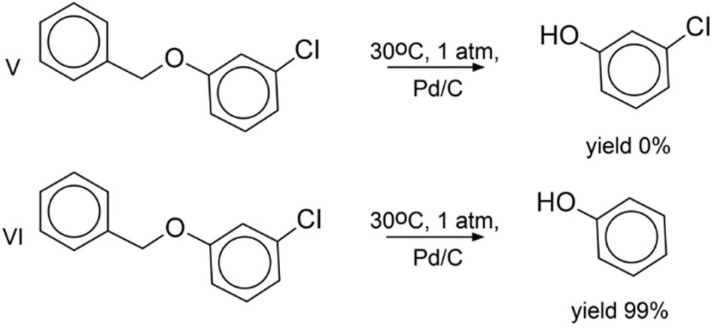
Regioselective reduction of 1-benzyloxy-3-chlorobenzene. The same conditions were predicted for reactions V and VI: medium t, low p, palladium on carbon. As a result, Reaction VI was carried out with the yield 99%, 1% was the incomplete reduction product—(phenoxymethyl)-benzene.

Experimental validation of the LRM approach was performed on hydrogenation reactions involving three compounds absent from the training set (Figure 2, Figure 3 and Figure 4). Each of them bears two groups reactive under hydrogenation conditions: the nitro and benzyl groups (Figure 2 and Figure 3), or the nitro group and chlorine (Figure 4). All possible products for three selected reactants were manually enumerated, resulting in 54 molecules, see Figures S9–S11 in Supplementary Materials. Most of the generated reactions were found implausible. For example, under hydrogenation conditions, the nitro group is more reactive than the benzyl group; therefore, one can hardly remove the benzyl group by leaving the nitro group intact. Aromatic ring hydrogenation requires harsh conditions leading to the reduction in all other groups. However, LRM predicts conditions for any reaction, even for an unfeasible one. To fix this shortcoming, the Fragment Control of the applicability domain was used. Reactions leading to a product found outside of the model AD were discarded.

As a result, only three products were found within the model’s AD (reactions I, III, IV). Three other reactions leading to products outside of the AD were added (II, V, VI) as challenging cases of selectivity. Notice that hydrogenation of 1-benzyloxy-3-chlorobenzene (reactions V and VI in Figure 4) lead to products outside of the AD, which contrasts with hydrogenation of 1-benzyloxy-4-nitrobenzene leading to products within the AD (reactions III and IV in Figure 3). For all studied reactions, only top-1 predicted conditions were selected. The reactions were carried out in a flow reactor under conditions predicted by our model. For all reactions within the AD (I in Figure 2 and III, IV in Figure 3), the dominant products detected with GCMS were those theoretically expected. By-products in the reactions were either the starting compounds or products of incomplete hydrogenation (Figure 2 and Figure 3). The latter might be explained by insufficient reaction time. In the case of reactions outside the AD, in 2 out of 3 cases, the target compound was also experimentally found as a dominant product (II, IV in Figure 2 and Figure 4, respectively). Interestingly, log-likelihood for the reactions V and VI (0.61 and 0.97, respectively) pointed out that conditions for the latter were predicted with higher confidence.

## 3. Computational Procedure

### 3.1. Data Curation

The initial dataset of one-step hydrogenation reactions containing 591,563 reactions (391,880 chemical transformations) was extracted from the Reaxys^®^ database in May 2019. We follow the same terminology as in our earlier publication [13]: by “transformation” we mean a set of reactants and products, “reaction” is a transformation carried out in the given conditions. Hydrogenation reactions were revealed by the presence of “H2” or “hydrogen” keyword in the reagent list for at least one condition corresponding to a reaction. Chemical structures were standardized according to the protocol described by Gimadiev et al. [13]. CGRtools [14] was used for functional group normalization, aromatization, removing explicit hydrogens and duplicate cleaning. ChemAxon Standardizer [15] was used for atom-to-atom mapping [16]. Molecules with irregular valences, radicals or unbalanced ions were removed from the dataset. Reactants that had atoms not mapped to any product atom were transferred to the reagents list as suggested in publication [13]. After standardization, the dataset included 566,896 reactions (345,619 chemical transformations).

Special attention was paid to conditions standardization, in which we generally followed a previously described procedure [9], which was improved by taking into account our cumulated experience. Detailed condition curation procedure is described in Supplementary Materials, Figures S1–S4.

First, advanced multistep/multistage reactions detection procedure were used to identify and remove reactions wrongly recorded as single-step ones. Values from ‘temperature’ and ‘pressure’ fields were converted to numeric. If ranges for these parameters were given, they were either averaged (if range spread was small enough) or removed. Moreover, the conditions from the text field ‘conditions’ have also been handled. All synonyms of room temperature and atmospheric pressure were replaced by 25 °C and 1 atm, respectively. Eventually, 96,755 reactions (corresponding to 68,711 transformations) annotated with both temperature and pressure were left for further consideration (Table 2).

The names of chemical agents were extracted from “reagents”, “catalysts” and “solvents” fields. Raw names of chemical agents were transformed to the standard names using the synonym dictionary described in our earlier publication [9]. For some 400 chemical agents, one standard name corresponds to more than one raw name. For example, the standard name “Raney nickel catalyst” matches 416 unique ‘raw’ names. Collected to date, the synonym dictionary contains raw names for 2323 standardized agents. This has been used to standardize the raw names of agents for more than 87% reactions in the initial dataset (all chemical agents for these reactions were standardized; see Table S1 in Supplementary Materials). Complex catalysts or metal catalysts and their supports were identified and one standard name was assigned. For example, “Lindlar catalyst” was used as a standard name for the raw names containing palladium on a specific support (calcium carbonate, barium sulfate, barium carbonate, calcium fluoride or activated carbon), together with a lead-containing compound and/or quinoline, and/or with a sulfur-containing compound [17,18,19,20]. Each organic compound was represented as unique SMILES string. Information about stereochemistry was excluded since it cannot be accounted for by the developed model. In such a way, the coverage of standardization (share of standardized names (cumulative frequency of occurrence) relative to the cumulative frequency of occurrence of the original names of chemical agents) was more than 95%.

Tags indicating a possible role of a compound in the hydrogenation reaction (e.g., “catalyst”, a “catalytic poison”, an “acid”, a “base”) were assigned to every standard name. Such an assignment was performed semi-automatically. For instance, a standard name was automatically tagged as “catalysts” if it contained at least one of the following atoms: Zr [21,22], Rh, Ir, Ru, Pt, Pd, Ni, Fe, Co, Os, Cu [23,24], Re [24], Ag [23,25], Au [23], W, Mo [26,27], Ti [28], Mn [29], In [30]. We found that such compounds were often recorded in the Reaxys^®^ database in the “reagents” field. This explains why the number of catalysts has increased after standardization (see Table 2).

Reactions without a standardized chemical agent or with more than one catalyst were discarded. The curated modeling dataset contained 66,840 reactions for 54,345 transformations, see Table 2.

### 3.2. Training/Test Set Preparation

In order to avoid a biased assessment of the model, the training and test sets did not contain the same reaction transformation. Thus, the reactions involving the same transformation that proceeded under different conditions were transferred either to training or test sets. Two external test sets—prospective and retrospective—were formed for a more complete assessment of the predictive performance of the developed model. The prospective test set was formed from standardized chemical transformations, which were reported for the first time in the literature source in 2017–2019, Table 3. The retrospective test set was formed by a random selection of 10% chemical transformations from a pre-standardized dataset after the formation of a prospective test set, Table 3. The training set resulted from subtraction of the reactions of retrospective and prospective test sets from the initial modeling set. The duplicates of formed condition bit-strings were removed. The size of resulting datasets is given in Table 3. The selection process guarantees that there is no intersection between both test sets and training set.

The procedure of training/test set splitting is also shown in Figure S5 (Supplementary Materials).

### 3.3. Descriptors

**Figure 5:**
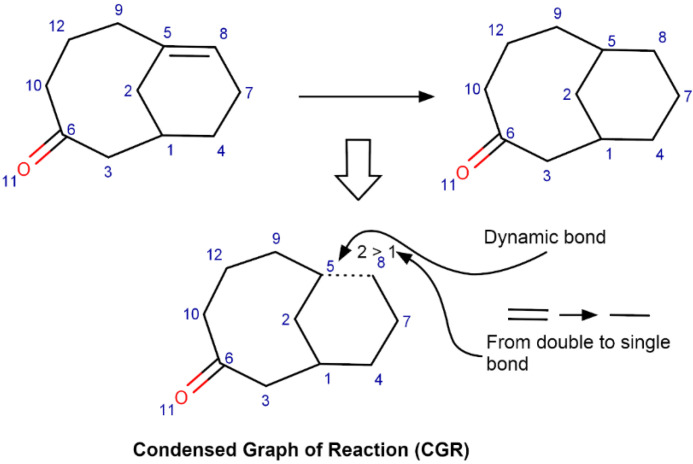
Condensed Graph of Reaction (CGR) generation.

The ISIDA fragment descriptors [31] were generated for each reaction encoded by Condensed Graph of Reaction (CGR) [31] using CGRtools library [14]. A CGR results from a superposition of related atoms of products and reactants. A CGR represents a reaction as a pseudomolecule described by both conventional and “dynamic” atoms and bonds. Dynamic bonds describe chemical transformations: the breaking or formation of chemical bonds, transformation of a single bond to a double bond, etc. (see Figure 5).

The ISIDA fragment descriptors were generated for CGRs using the ISIDA Fragmentor 2017 software [32] wrapped by an in-house Python CIMtools library [33]. Atom-centered fragments based on sequences of atoms and bonds of fixed length ranged from two to four were used. Additional options for descriptor generation were applied, such as Formal Charge encoding and all fragments formation, so fragments could either contain dynamic bonds or not. For the training set, more than 181,000 descriptors were generated. The rare fragments with a frequency less than five were removed leading to the reduced pool of 28,022 descriptors. Further dimensionality reduction was performed with the help of Incremental Principal Component Analysis [34], implemented in the scikit-learn package [35]. Finally, 1500 principal components were retained, which corresponds with the explained variance of 96.5%. 

**Figure 6:**
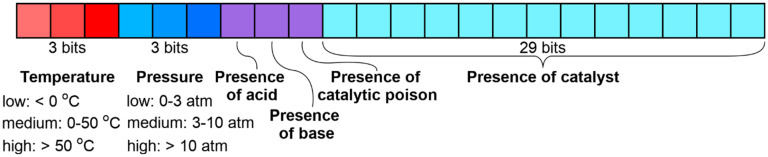
Conditions components representation.

Reaction conditions were encoded by a 38-bit bit vector describing the following condition components: temperature, pressure, additives, and catalysts, as shown in Figure 6. Three bits per parameter were allocated for temperature and pressure. They encode ranges of low (<0 °C), medium (0–50 °C) and high (>50 °C) temperature, and low (0–3 atm), medium (3–10 atm) and high (>10 atm) pressure. The presence of additives was also binned by 3 bits—for acid, base, and catalytic poison. Catalysts were encoded by 29 bits for the most common catalysts used in >100 reactions from the training set. A list of considered catalysts is given in Appendix A4 of Supplementary Materials.

In the bins corresponding to the temperature, pressure, and catalyst, only one bit can be activated, whereas acid, base, or catalytic poison could be present simultaneously. As a result, 2088 possible unique combinations of condition components can be identified. However, in reality, only 507 unique combinations of condition components were found in the Modeling set (Table 3).

### 3.4. Methods

#### 3.4.1. Likelihood Ranking Model

A Likelihood Ranking Model (LRM) is based on a feed-forward artificial network trained in an end-to-end fashion, implemented with the help of Keras library [36] with Tenforflow [37] backend. 1500 descriptors encoding CGR were used in the input layer; the output layer had sigmoid activation and predicted probabilities $\hat{Y}$ for 38-bit vector of conditions described in Figure 6. The optimal architecture was designed using the MRR@5 metric (see below) as a maximization criterion calculated in 5-fold cross-validation with a single repeat. The following hyperparameters were optimized:

- the number and size of hidden layers;
- the number of layers: 1–3;
- the number of neurons: 10, 20, 40, 80, 100, 200, 400, 800 (for 1–3 layered networks, the upper hidden layer does not have more neurons than the previous one), 1000, 2000, 3000, 4000 (for networks with a single hidden layer);
- the number of learning epochs: 10–500;
- the dropout value: 0–0.5 (for 2–3 layered networks only).

The best model architecture had a single hidden layer with 2000 neurons and ReLU activation function, trained in 200 epochs. The Adam optimizer was applied, the learning rate was set to 0.001. At every next epoch the latter was updated via multiplication by a factor of 0.99.

**Figure 7:**
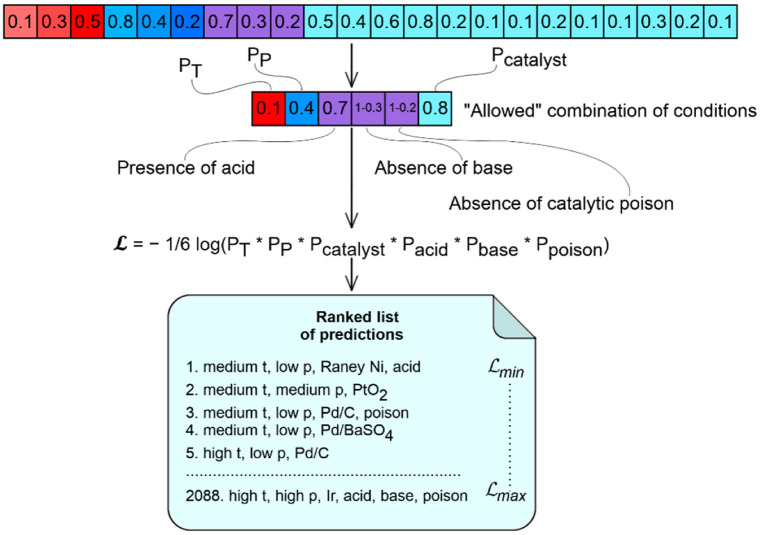
Example of the model predictions for a given chemical transformation. The model outputs the 38-dimentional vector corresponding to condition components shown in Figure 6. This vector is used to derive 2088 allowed combinations of conditions, each corresponding to particular temperature, pressure, catalyst, and presence of acid, base and catalytic poison. The list of allowed condition combinations is ranked according to the likelihood ℒ.

We ranked possible combinations of condition components according to the log-likelihood value ℒ. The latter was calculated as the negative logarithm of the geometric mean of probabilities $p_{i}^{temp}$, $p_{i}^{pres}$, $p_{i}^{cat}$, $p_{i}^{acid}$, $p_{i}^{base}$, $p_{i}^{poison}$, see Figure 7. Probabilities for temperature $p_{i}^{temp}$, pressure $p_{i}^{pres}$ and catalysts $p_{i}^{cat}$ correspond to selected temperature/pressure/catalyst bins. Since additives (acid, base, catalytic poison) can either be present and absent, their probabilities for the *i*th reaction were taken from the Bernoulli distribution $p_{i}^{j\epsilon \left\{acid,\;base,poison\right\}}={\left(P_{i}^{j}\;\right)}^{{\hat{Y}}_{i}^{j}}{\left(1-P_{i}^{j}\right)}^{1-{\hat{Y}}_{i}^{j}}$, where ${\hat{Y}}_{i}^{j}$ is set to 1 if acid, base, or catalytic poison is selected in condition components combination, otherwise, it is 0, $P_{i}^{j}\;$ is the probability corresponding to acid, base, or poison. Combinations of condition components were ranked in ascending order with respect to ℒ; those with higher probability were considered more suitable for a given reaction.

#### 3.4.2. Null Model

The null model, used as a negative control and returns a combination of condition components corresponding to their frequency in the training set. For any transformation, the null model returns the same ranked list of conditions, regardless of the chemical structures of the reactants; see Table S2 of Supplementary Materials.

#### 3.4.3. k Nearest Neighbors Approach

Ranking k Nearest Neighbors approach (kNN) was used as a simple alternative for the proposed neural network model. A similarity-based approach was also applied in our previous publication [9]. Gao et al. [7] reported that a similar nearest neighbor approach performs comparably to the neural network model, however is more computationally demanding.

**Figure 8:**
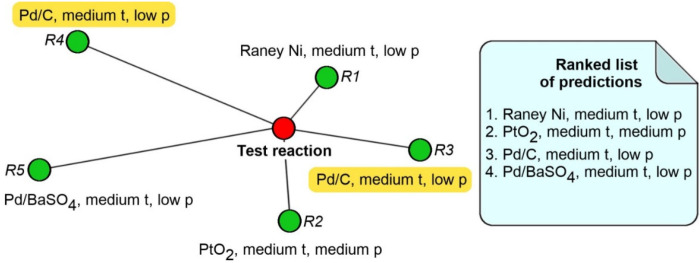
Conditions prediction by the k Nearest Neighbors approach. Note that the conditions are the same for reactions R3 and R4, thus conditions combination for more distant reaction R4 was ignored.

In the kNN approach, for a given query reaction, the most similar objects were retrieved from the training set. As a result, the model returns conditions corresponding to these training set reactions, sorted according to the similarity to the query (Figure 8). If the same condition combinations appeared several times in the returned list, the conditions of the more far-lying reaction were ignored. A pairwise reactions similarity was assessed as Euclidian distance calculated using CGR-based fragment descriptors compressed with the help of Incremental Principal Component Analysis [34] (see above).

#### 3.4.4. Model from ASKOS

For the comparison purpose, we used the model proposed by Gao et al. [7], which is available on Github [38] as part of the ASKOS system. This neural network (hereafter denoted as the ASKOS model) sequentially predicts catalyst, first solvent, second solvent, first reagent, second reagent, and temperature as a continuous value. The returned condition combinations list contains up to 18 entries, enumerated from the list of the best two catalysts, three first solvents, one second solvent, three first reagents, and one second reagent. The model inputs SMILES strings of reactions. For six reactions from the retrospective test set, the SMILES reading step has failed.

Since the output of the ASKOS model is represented as Reaxys^®^ chemical ID, SMILES or compound name, it requires the name standardization procedure. Therefore, the protocol of ‘raw’ names standardization and tags generation (Supplementary Materials, Figures S3 and S4) was applied for predicted catalysts, solvents, and reagents. Compounds missing from our dictionary of raw and standard names were added manually. Thus, 1592 new “raw” names were added for the test sets. The procedure for a bit-string conversion was the same as described in the Data Preparation section.

#### 3.4.5. RecNN

The ASKOS model from Gao et al.’s paper [7] was trained on a diverse data set (~10 million examples from Reaxys^®^), which includes various types of reactions. In order to benchmark neural network architecture by Gao et al. [7] with the ones used in this study, the former was trained on our training set containing catalytic hydrogenation reactions only. The tool of Gao et al. was slightly modified in order to obtain the output similar to that of our model. Thus: (i) an additional layer predicting pressure was added; and (ii) temperature and pressure were predicted as categorical variables as described in Section 3.4.1. By this reasoning, softmax cross-entropy loss was used instead of mean squared error. We kept the restrictions related to the number of predicted conditions as in the original publication [7]: only the 2 best catalysts were used to predict the remaining condition components. For clarity, this model is called RecNN since it predicts conditions recurrently.

### 3.5. Model Performance Assessment

Standard metrics for machine-learning model quality assessment do not allow one to evaluate the predictive ability of our model based on the reaction conditions ranking approach.

Therefore, two metrics used in the information retrieval domain [39] for the analysis of ranked outputs were used instead.

The first one, *Mean Reciprocal Rank at K* (*MRR@K*) [39] was used for the hyperparameter optimization of the Likelihood ranking model. MRR@K is the arithmetical mean of the inverse of the best experimental rank (Equation (1)).

(1) $$MRR@K=\frac{1}{N}\underset{i}{\sum }\frac{1}{\min\left(R_{i}\left(K\right)\right)}$$

where $R_{i}=\left\{r_{i}\right\}-$ list of ranks of relevant items (corresponding to experimental condition components combinations) within the top *K* of considered ranks, *N*—number of considered reactions. *MRR@K* varies in the range [0, 1]. An example of the *MRR@K* calculation is given in Figure S6 in Supplementary Materials. The *MRR@K* was chosen as: firstly, the metric estimates how often conditions are predicted correctly; secondly, it takes into account the position of the elements.

Another metric, *Precision at K* (*P@K*), was used for results interpretation. *P@K* is the percentage of reactions in which at least one of the experimental condition components combinations were found within the top *K* of predicted conditions combinations (see Equation (2)).

(2) $$P@K=\frac{n@K}{N}*100\%$$

### 3.6. Applicability Domain

By default, the applicability domain (AD) of the models was not considered unless specifically mentioned. In that case, the Fragment Control approach [33] was applied. A given reaction was considered out of a model’s AD if its CGR contained a fragment absent in the training set CGRs. All fragments were enumerated using ISIDA fragment descriptors. Fragment Control is implemented in the in-house library CIMtools [33] and based on ISIDA Fragmentor [32].

### 3.7. Experimental Validation

All reactions were carried out in a ThalesNano H-Cube Pro™ flow reactor.

Materials. Solvents of chemically pure and analytical grades were purified according to known procedures [40] before use. All benzyl ethers were synthesized using known procedure [41]. NMR spectra of 1-(benzyloxy)-4-chlorobenzene, 1-(benzyloxy)-4-nitrobenzene [42] and 1-(benzyloxy)-3-nitrobenzene [43] agree with those reported in the literature. As catalysts, 10% palladium on carbon (THS-01141), Raney nickel (THS-01142), and 10% platinum on carbon (THS-01143) from Thales Nano were used.

Experiments. A vial with a semipermeable membrane was charged with 10 mL of solution of a corresponding benzyl ether with a concentration of 0.01 M (10 mL) in absolute tetrahydrofuran (with the addition of 1% volume of glacial acetic acid if necessary), and the vial was placed in an automated sample feeder of ThalesNano H-Cube™. The catalyst cartridges were mounted in the 6-channel CatCart Changer™. The solution was passed through the reactor with automated sampling, flow rate 0.5 mL/min, and then collected by an automated sample feeder. After that samples were analyzed by GCMS.

GCMS. Gas chromatography-mass spectrometry analysis was performed on a Shimadzu GCMS-QP2010 Ultra instrument using: 0.25 μm 30 m HP-5MS capillary column; carrier gas He, flow rate 2 mL/min, split ratio 40; injector temperature 250 °C; oven gradient temperature rising from 70 to 140 °C with the rate of 10 deg/min, a 2 min hold at 140 °C and a gradient rising from 140 to 250 °C with the rate of 10 deg/min; MS TIC mode scanning ions in the range 35–400 *m*/*z*.

## 4. Conclusions

Reaction condition prediction is quite a challenging task due to several reasons: (i) a particular reaction may proceed under several different conditions; (ii) all conditions leading to a particular product are never explored experimentally; and (iii) negative data are usually absent. Here, we demonstrate that consideration of reaction condition prediction as a ranking problem solves all aforementioned problems. A simple and efficient likelihood ranking modelling approach for reaction condition prediction was proposed. Being trained on a dataset of catalytic hydrogenation reactions extracted from Reaxys^®^, LRM outperformed some popular approaches used for condition prediction such as k Nearest Neighbors and recurrent prediction of reagents, solvents, catalysts, and temperature using an artificial neural network of a complex architecture [7].

The ability of the model to propose conditions leading to the desired product was tested experimentally on a challenging set of six reactions with rich selectivity issues. Only top-1 predicted condition was selected for the experimental check. The experiment confirmed a good predictive ability of the model: conditions predicted for five out of six reactions led to the desired product. The remaining reaction was outside of the model applicability domain, which confirms the importance of an AD usage in the model application.

Notice that the likelihood ranking can be applied only if all possible condition combinations can be enumerated. This limits the applications for diverse reactions datasets where possible condition space is extremely vast. In this case, the nearest neighbor approach seems to be a reasonable alternative to LRM as it does not require condition combinations enumeration. However, for large training datasets, the speed drastically decreases and some special approaches (e.g., FAISS [44,45]) are required to solve this problem. For such cases, neural networks with constrained ranking of possible condition combinations seem to offer a good alternative.

## Figures and Tables

**Table 1:** Percentage of cases for which at least one of experimental condition component combinations were found within the top of a sorted list of predicted conditions (retrospective test set). LRM—likelihood ranking model, NM—null model.

Mode	TOP 1		TOP 3		TOP 5		TOP 10	
	LRM	NM	LRM	NM	LRM	NM	LRM	NM
catalyst	82	66	93	75	96	85	98	91
pressure	78	59	100	100	100	100	100	100
additives	91	87	99	97	99	100	99	100
temperature	93	85	100	100	100	100	100	100
catalyst, pressure	67	44	85	66	90	71	95	81
additives, pressure	72	52	93	87	97	92	99	98
catalyst, additives	76	58	89	67	92	76	96	84
catalyst, pressure	67	44	85	66	90	71	95	81
catalyst, temperature	78	60	90	70	93	76	96	86
temperature, pressure	75	56	93	85	98	97	100	100
catalyst, pressure, additives	63	39	80	58	86	64	91	73
catalyst, temperature, additives	73	53	86	61	89	69	93	80
catalyst, temperature, pressure	65	42	81	60	86	65	91	75
temperature, pressure, additives	69	50	88	74	93	84	97	94
catalyst, additives, pressure, temperature	61	38	77	53	82	59	88	67

LRM—Likelihood ranking model, NM—null model.

**Table 2:** Statistics of conditions standardization (number of transformations/reactions).

	Before Standardization	After Standardization
Initial dataset	345,619/566,896	
Includes temperature	175,697/295,331	140,843/208,671
Includes pressure	150,100/215,191	113,057/151,154
Includes catalysts	207,070/275,546	279,739/419,223 *
Includes temperature and pressure	93,769/143,138	68,711/96,755
Includes temperature, pressure and catalysts	36,684/52,367	57,427/72,871 *
Modeling dataset (T, P and single catalyst)	54,345/66,840	

* Number of catalysts increased after standardization since in initial dataset many catalysts were recorded as reagents.

**Table 3:** Dataset statistics.

Dataset	Number of Reactions	Number of Transformations	Number of Unique Combinations of Condition Components
Modeling set	66,840	54,345	507
Training set	42,541	40,870	494
Retrospective test set	5103	4888	264
Prospective test set	5011	4992	144

The modeling sets cannot be provided being the proprietary data of Reaxys^®^.

## Data Availability

The data are not publicly available due to being the proprietary data of Reaxys^®^.

## Supplementary Materials

The supporting information can be downloaded at: https://www.mdpi.com/article/10.3390/ijms23010248/s1.

## Abbreviations

kNN	k nearest neighbor approach
AD	applicability domain
QSAR	quantitative structure-activity relationship
LRM	Likelihood ranking model
NM	null model
