# Supplementary material for: Prediction of Optimal Conditions of Hydrogenation Reaction Using the Likelihood Ranking Approach
Source: Int J Mol Sci. 2021 Dec 27;23(1):248. doi: 10.3390/ijms23010248 (PMC8745269; doi:10.3390/ijms23010248)

Supporting Information

Prediction of Optimal Conditions of Hydrogenation Reaction using the Likelihood Ranking Approach

Valentina A. Afonina[1], Daniyar A. Mazitov[1], Albina Nurmukhametova[1], Maxim D. Shevelev[1,2], Dina A. Khasanova[1], Ramil I. Nugmanov[1], Vladimir A. Burilov[1], Timur I. Madzhidov[1,*], Alexandre Varnek[2,3,*]

[1] Chemoinformatics and Molecular Modelling Lab, A.M. Butlerov Institute of Chemistry, Kazan Federal University, Kremlyovskaya str. 18, 420008 Kazan, Russia; ValAAfonina@kpfu.ru (V.A.A.); DaniAMazitov@kpfu.ru (D.A.M.); albinka2491@mail.ru (A.T.N.); mdshev7@gmail.com (M.D.S.); DAHasanova@stud.kpfu.ru (D.A.K.); nougmanoff@hotmail.com (R.I.N.); Vladimir.Burilov@kpfu.ru (V.A.B.)

[2] Laboratory of Chemoinformatics (UMR 7140 CNRS/UniStra), Université de Strasbourg, 4, rue Blaise Pascal, 67000 Strasbourg, France

[3] Institute for Chemical Reaction Design and Discovery (WPI-ICReDD), Hokkaido University, Kita 21 Nishi 10, Kita-ku, Sapporo 001-0021, Japan

* Correspondence: timur.madzhidov@kpfu.ru (T.I.M.); varnek@unistra.fr (A.V.)

Table of Contents

Appendix A1. Condition curation procedure....3

Figure S1. Scheme of temperature standardization....3

Figure S2. Scheme of pressure standardization....4

Figure S3. Standardization scheme of 'raw' names of chemical agents 5

Figure S4. Raw and standardized names and tags of chemical agents....6

Appendix A2. Additional reaction conditions curation results....7

Table S1. Statistics for synonyms dictionary....7

Appendix A3. Training / test sets preparation 8

Figure S5. Training / test datasets splitting scheme....8

Appendix A4. List of the most common catalysts in the training set 9

Appendix A5. Null model statistics....10

Table S2. Top-ten most frequent conditions in the training set 10

Appendix A6. Mean Reciprocal Rank calculation 11

Figure S6. Example of the Mean Reciprocal Rank calculation 11

Appendix A7. Additional validation of the performance of the models................12
Figure S7. Cumulative histogram of the P@K on training set in 5-fold cross-validation vs K 12
Figure S8. Cumulative histogram of the P@K on test sets in applicability domain vs K 12
Appendix A8. List of all possible products, manually generated for 1-benzyloxy-3-nitrobenzene, 1-benzyloxy-3-chlorobenzene, and 1-benzyloxy-4-nitrobenzene 13
Figure S9. All possible products, manually generated for 1-benzyloxy-3-nitrobenzene 13
Figure S10. All possible products, manually generated for 1-benzyloxy-3-chlorobenzene 14
Figure S11. All possible products, manually generated for 1-benzyloxy-4-nitrobenzene 15

Appendix A1. Condition curation procedure

Figure S1. Scheme of temperature standardization

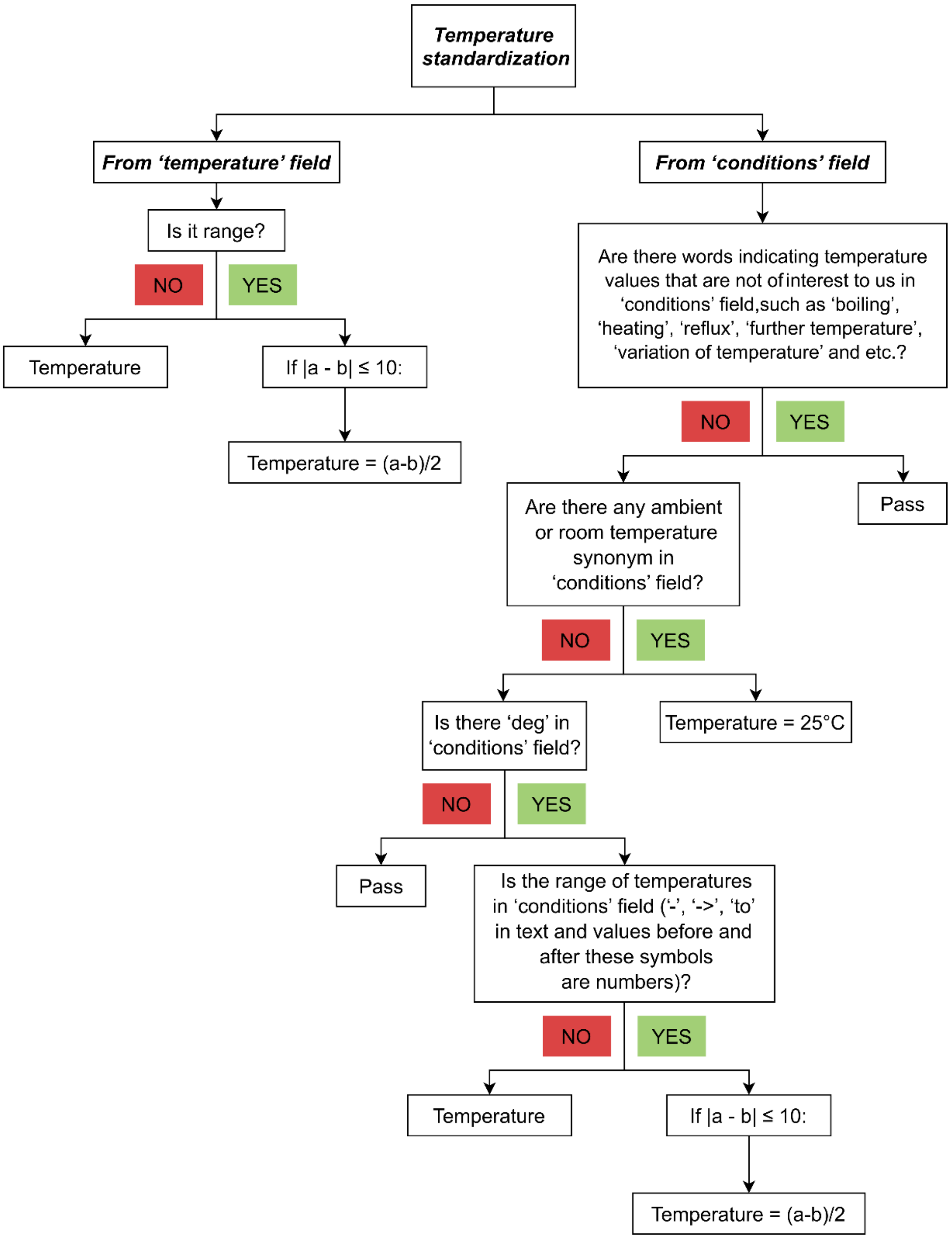

Figure S2. Scheme of pressure standardization

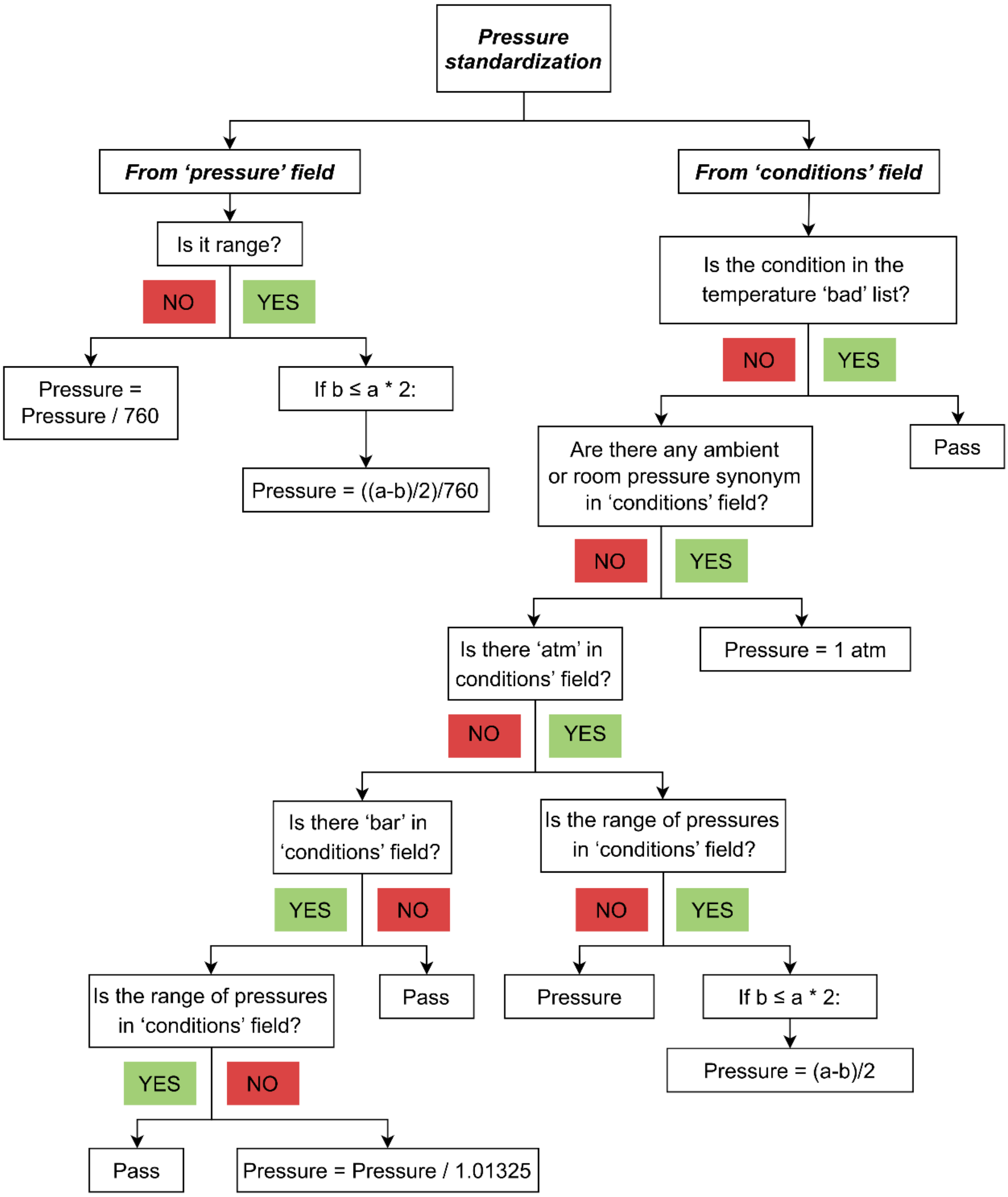

Figure S3. Standardization scheme of 'raw' names of chemical agents

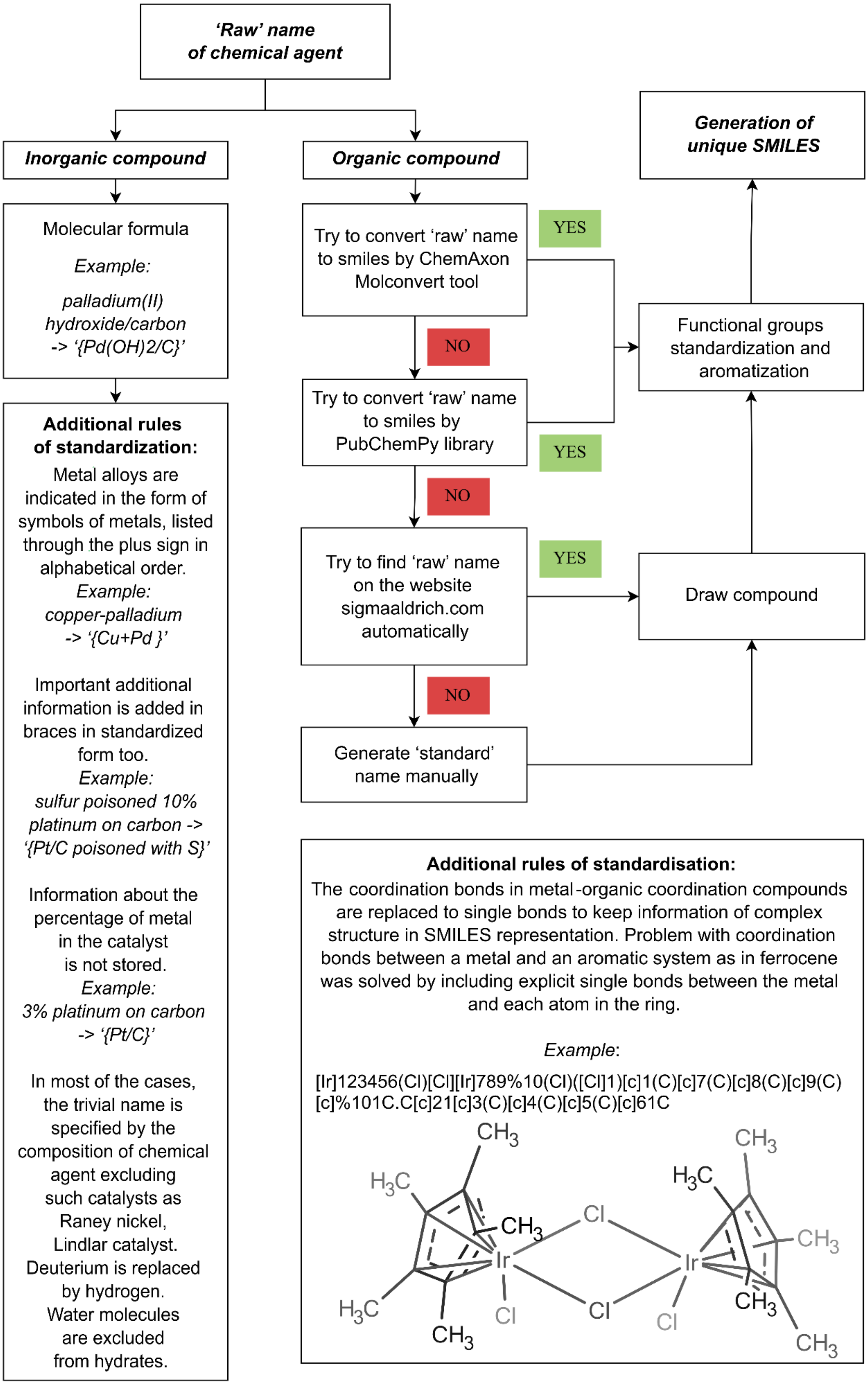

Figure S4. Raw and standardized names and tags of chemical agents

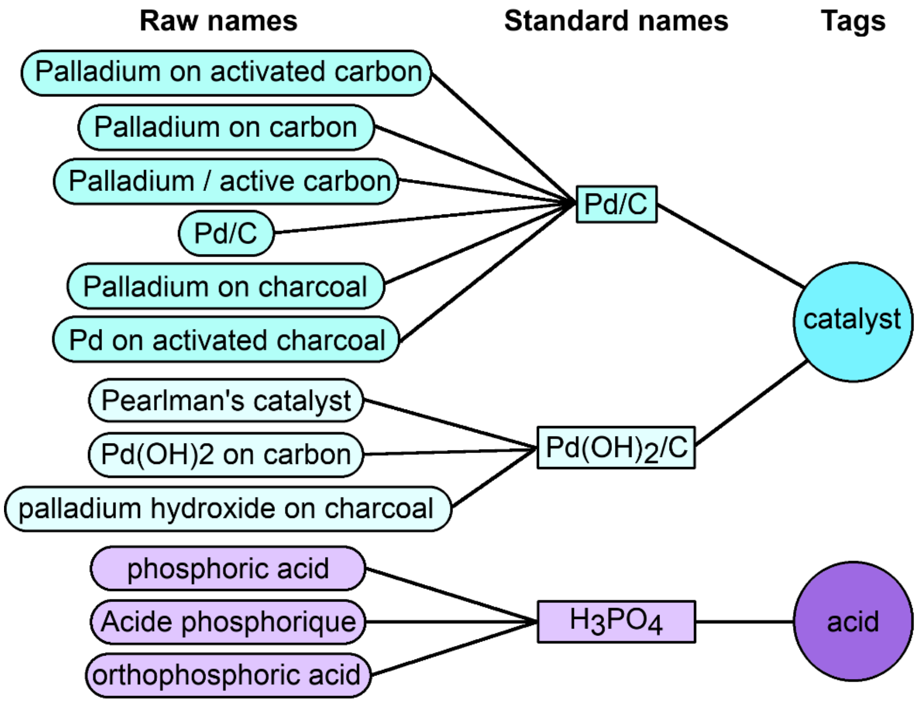

Appendix A2. Additional reaction conditions curation results

Table S1. Statistics for synonyms dictionary

Number of unique "raw" names in the initial dataset	28464
Number of unique standard names in the initial dataset	2323
Number of unique standardized "raw" names	4408
Number of standard names for which the tags were added	1125
Number of added tags	1235
Number of unique catalyst standard names	662
Maximum number of 'raw' names for one standard name before standardization	416
Maximum number of 'raw' names for one catalyst	416
Maximum number of 'raw' names for one any other chemical agents	10 [methanol]
Number of reactions for which all names of chemical agents were standardized in initial dataset	495011, 87.32%
Number of reactions for which at least one name of chemical agents was standardized in initial dataset	561880, 99.11%
Number of reactions for which only one name of chemical agents was not standardized	67097
Number of reactions for which all names of chemical agents were not standardized	2936
Coverage of standardization (frequency of standardized names / frequency of raw names)	95.61%

Appendix A3. Training / test sets preparation

Figure S5. Training / test datasets splitting scheme

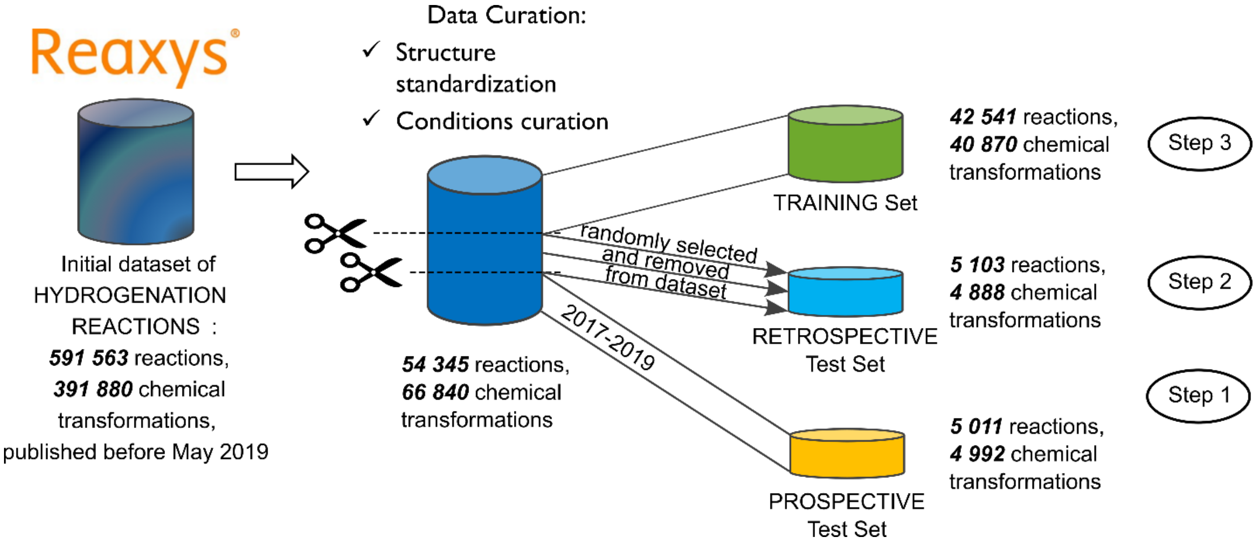

Appendix A4. List of the most common catalysts in the training set

1. Nickel catalyst (unknown type)
2. Raney nickel catalyst
3. Lindlar catalyst
4. Palladium(II) acetate
5. Palladium hydroxide(II) on carbon
6. Palladium hydroxide(II)
7. Palladium on aluminium oxide
8. Palladium on barium sulfate
9. Palladium on carbon
10. Palladium(II) chloride
11. Palladium catalyst (unknown type)
12. Platinum on aluminium oxide
13. Platinum on carbon
14. Platinum oxide(IV)
15. Platinum catalyst (unknown type)
16. Chloro(1,5-cyclooctadiene)rhodium (I) dimer
17. Dicarbonyl(acetylacetonato)rhodium(I)
18. Dirhodium tetraacetate
19. Tris(triphenylphosphine)rhodium(I) chloride
20. Bis(1,5-cyclooctadiene)rhodium(I) tetrafluoroborate
21. Tris(triphenylphosphine)rhodium carbonyl hydride
22. Bis(1,5-cyclooctadiene)rhodium(I) trifluoromethanesulfonate
23. 1,2-bis-[2,5-diethylphopholano] benzene(cyclooctadiene) rhodium(I) trifluoromethanesulfonate
24. Rhodium on aluminium oxide
25. Rhodium on carbon
26. Ruthenium on carbon
27. Bis(1,5-cyclooctadiene)diiridium(I) dichloride
28. Dicobalt octacarbonyl
29. Copper catalyst (unknown type)

Appendix A5. Null model statistics

Table S2. Top-ten most frequent conditions in the training set

Rank	Temperature[a]	Pressure[b]	Additives[c]	Catalyst	Percent of reactions in the training set
1	Medium	Low	-	Palladium on carbon	35.3
2	Medium	Medium	-	Palladium on carbon	13.6
3	Medium	High	-	Palladium on carbon	3.0
4	Medium	Low	-	Palladium hydroxide on carbon	2.4
5	Medium	Low	Acid	Palladium on carbon	2.3
6	High	High	-	Palladium on carbon	2.1
7	Medium	Low	-	Platinum(IV) oxide	2.0
8	High	High	-	Unknown nickel catalyst	1.8
9	High	Medium	-	Palladium on carbon	1.7
10	Medium	Low	-	Unknown nickel catalyst	1.7

[a] Temperature: low - less 0 °C , medium - 0-50 °C , high - more 50 °C

[b] Pressure: low - 0-3 atm, medium - 3-10 atm, high - more 10 atm

[c] Additives: "-" – the absence of any additive; acid, poison, base – the presence of an appropriate additive; other additives – the presence of any other additives except the acid, base or catalyst poison

The total number of unique combinations of conditions in the training set is 759.

Appendix A6. Mean Reciprocal Rank calculation

Figure S6. Example of the Mean Reciprocal Rank calculation

Reaction 1

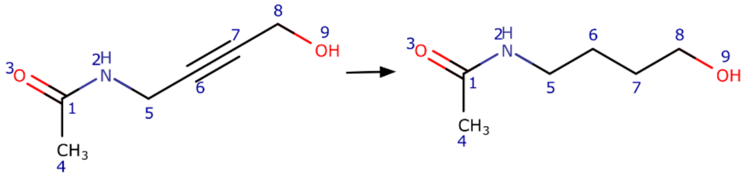

Reaction ID = 1108621

Sorted list of the predicted condition components combinations for Reaction 1:

1. Medium temperature, low pressure, unknown nickel catalyst, acid
2. Medium temperature, low pressure, unknown nickel catalyst
3. High temperature, high pressure, palladium on carbon
4. Low temperature, low pressure, palladium on carbon, base
5. Medium temperature, high pressure, unknown nickel catalyst, base

rank of experimental reaction conditions

real experimental data from literature

R_1 = {2}

Reaction 2

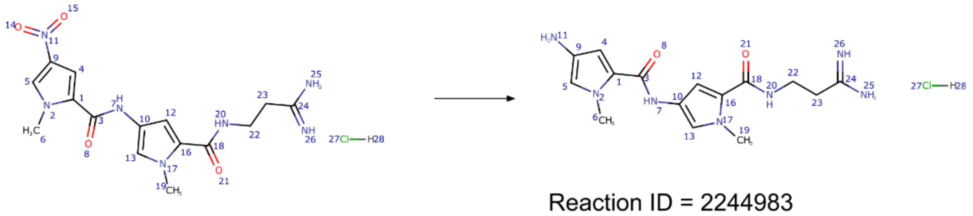

Reaction ID = 2244983

Sorted list of the predicted condition components combinations for Reaction 2:

1. High temperature, low pressure, palladium on carbon
2. Medium temperature, low pressure, unknown nickel catalyst
3. Medium temperature, low pressure, palladium on carbon
4. Low temperature, low pressure, palladium on carbon, base
5. Medium temperature, high pressure, unknown nickel catalyst, base

R_2 = {1, 3}

$$MRR@K = \frac{1}{N}\sum_{i}\frac{1}{min(R_i(K))}$$

Mean Reciprocal Rank for two reactions - Reaction 1 and Reaction 2 - at K = 3 will be calculated by formula above.

N = 2 (two reactions)

R_i is the list of ranks of relevant items (corresponding to experimental reaction conditions)

For Reaction 1:

R_1 = {2}; at K = 3 (max used rank = 3) R_1 = {2}

For Reaction 2:

R_2 = {1, 3}; at K = 3 R_2 = {1, 3}

$$\text{MRR@3} = \frac{1}{2} * (\frac{1}{2} + \frac{1}{\min(1, 3)}) = 0.75$$

Appendix A7. Additional validation of the performance of the models

Figure S7. Cumulative histogram of the P@K on training set in 5-fold cross-validation vs K

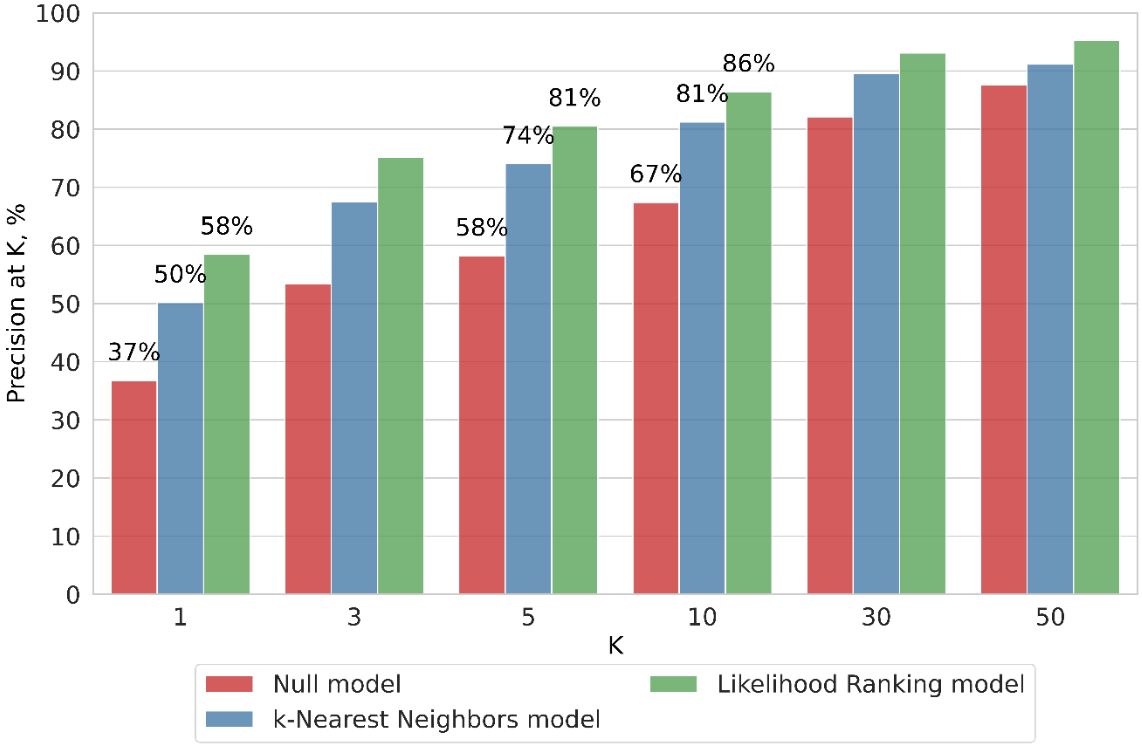

Figure S8. Cumulative histogram of the P@K on test sets in applicability domain vs K

a, b – retrospective test set, c, d – prospective test set; a, c – pressure is ignored

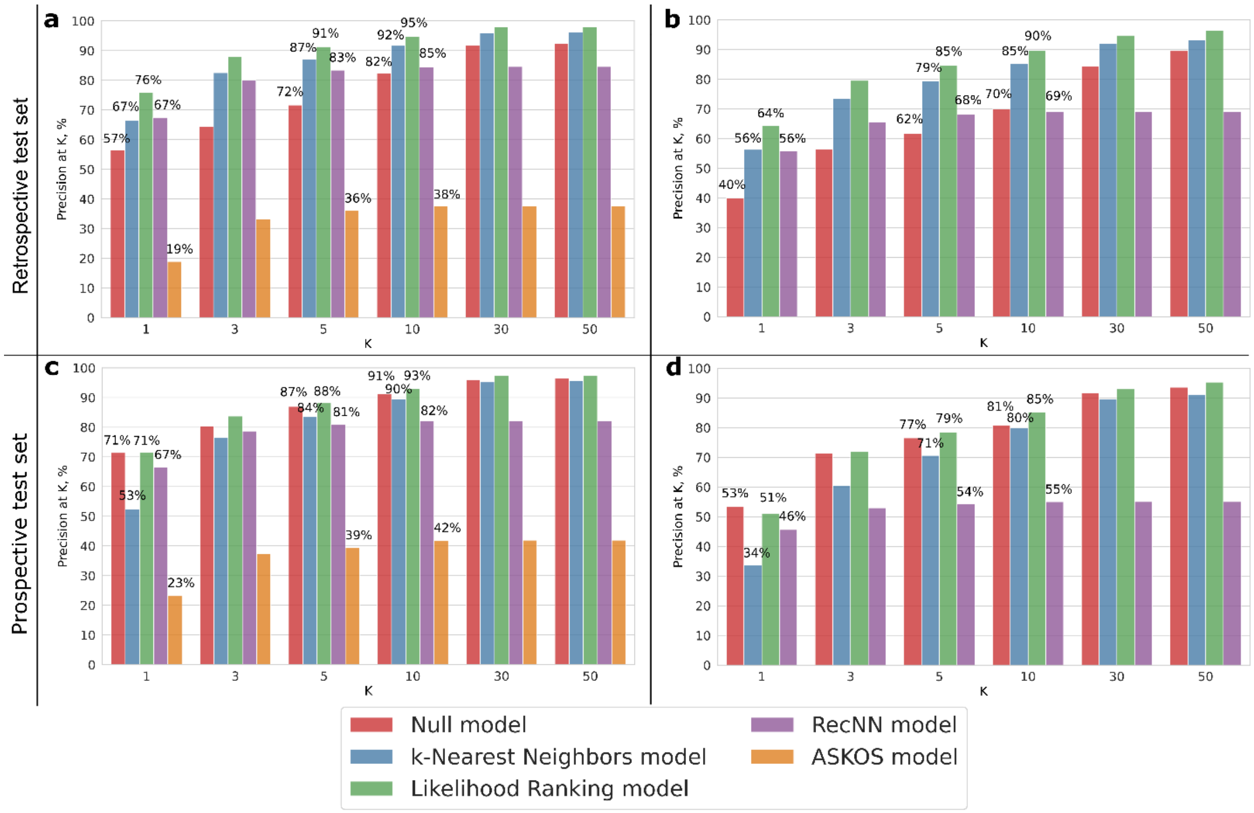

Appendix A8. List of all possible products, manually generated for 1-benzyloxy-3-nitrobenzene, 1-benzyloxy-3-chlorobenzene, and 1-benzyloxy-4-nitrobenzene

Figure S9. All possible products, manually generated for 1-benzyloxy-3-nitrobenzene

Product in the applicability domain is surrounded by a green frame

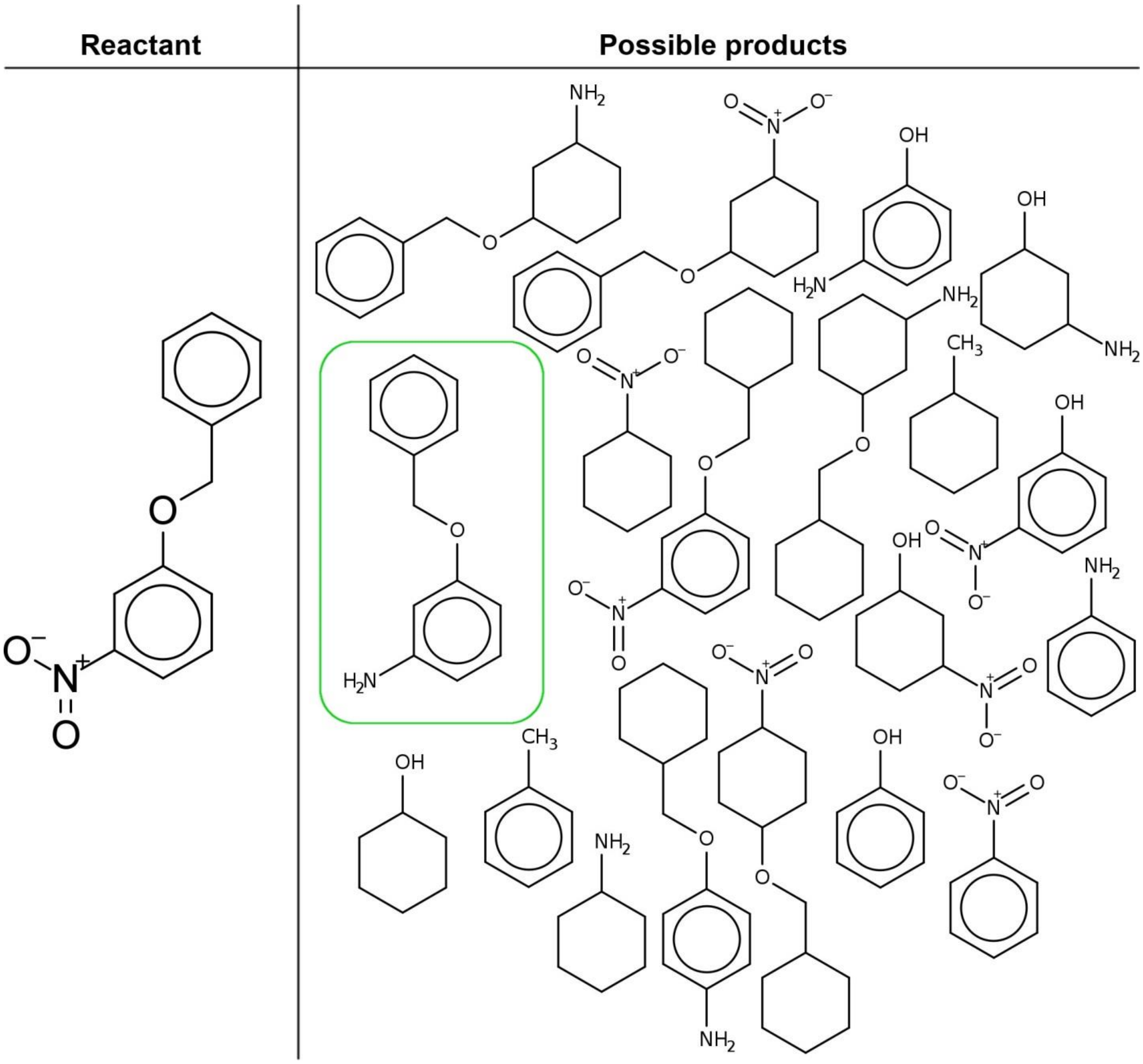

Figure S10. All possible products, manually generated for 1-benzyloxy-3-chlorobenzene

All products are outside the applicability domain

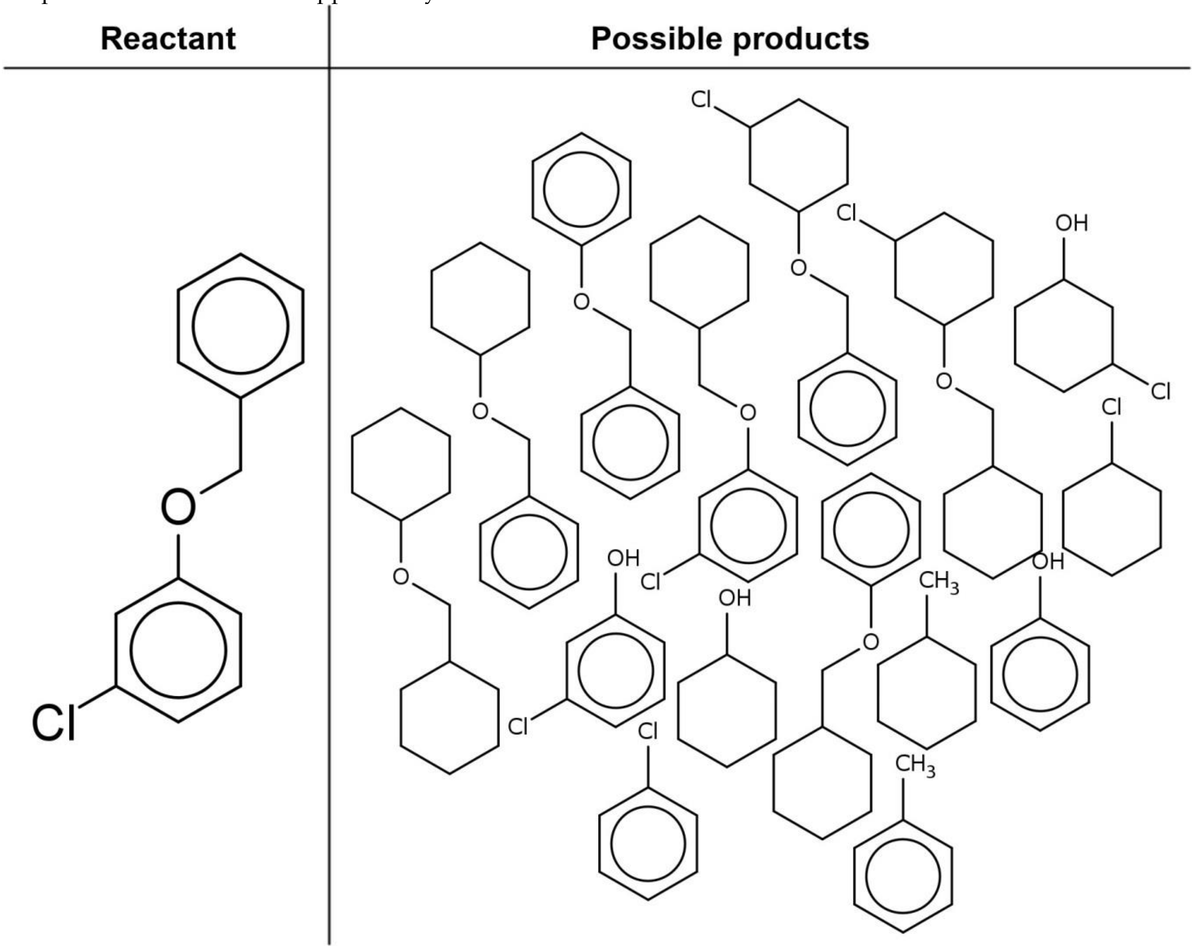

Figure S11. All possible products, manually generated for 1-benzyloxy-4-nitrobenzene

Products in the applicability domain are surrounded by a green frame

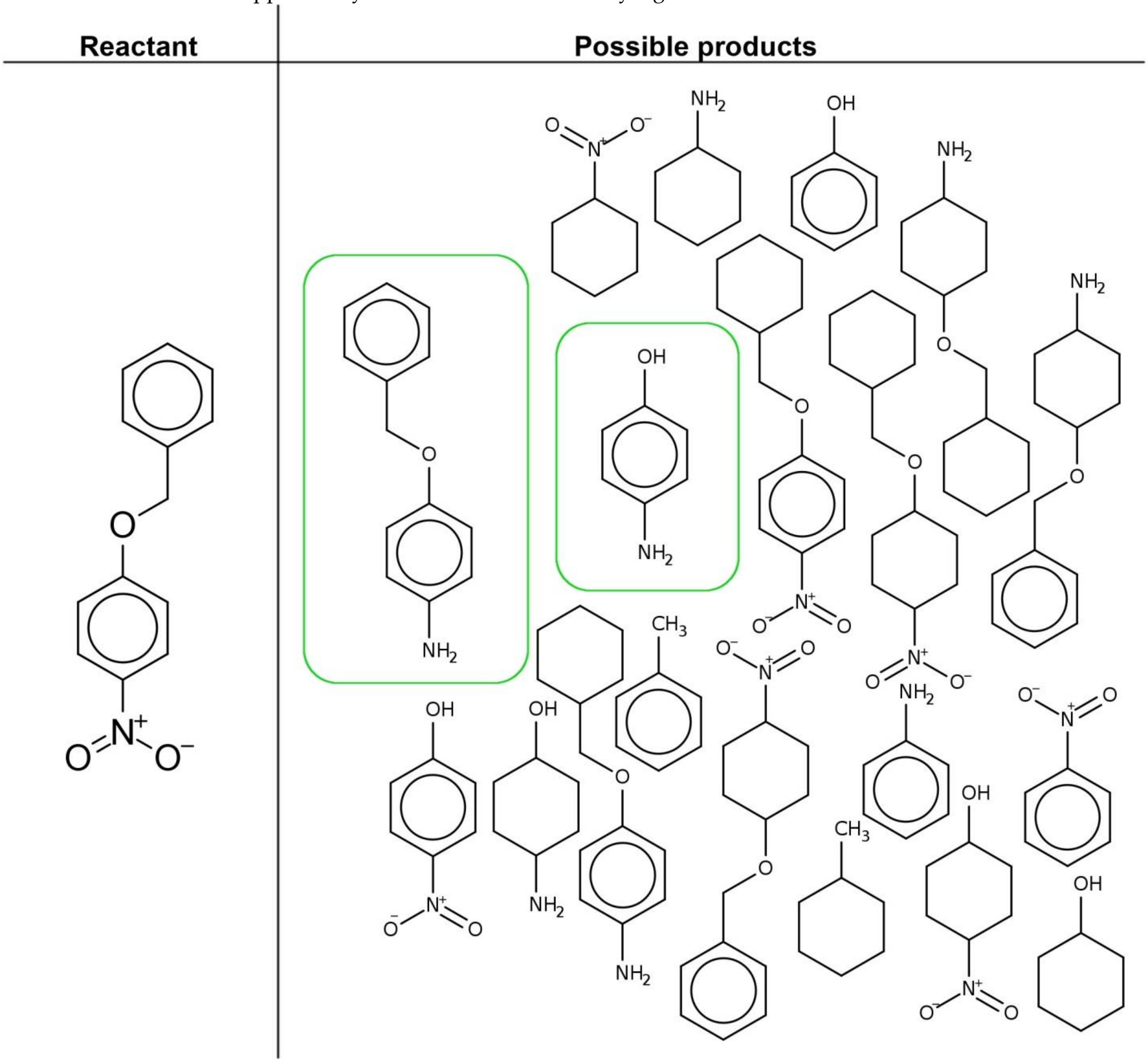

Supplement: Supplementary file 1 [file ijms-23-00248-s001.zip › ijms-1500215-supplementary.pdf]
